# Supplementary material for: Amino Acid-Metabolizing Enzymes in Advanced High-Grade Serous Ovarian Cancer Patients: Value of Ascites as Biomarker Source and Role for IL4I1 and IDO1
Source: Cancers (Basel). 2023 Jan 31;15(3):893. doi: 10.3390/cancers15030893 (PMC9913486; doi:10.3390/cancers15030893)
Supplement: Supplementary file 1 [file cancers-15-00893-s001.zip › cancers-2042352-supplementary.pdf]

Supplementary Materials

Amino acid-metabolizing enzymes in advanced high-grade serous ovarian cancer patients: value of ascites as biomarker source and role for IL4I1 and IDO1

Yvonne Grobben, Judith E. den Ouden, Cristina Aguado, Anne M. van Altena, Aletta D. Kraneveld and Guido J. R. Zaman

Table S1. List of analytes and isotope-labelled internal standards used for LC-MS/MS analysis.

Analyte	Catalog Number	Supplier	Internal Standard	Catalog Number	Supplier
L-Arginine·HCl	105000250	Acros Organics	L-Arginine-d7·HCl	A769501	Toronto Research Chemicals
L-Ornithine·HCl	12.956.55	Janssen	L-Ornithine-d6·HCl	O695553	Toronto Research Chemicals
L-Citrulline	C7629	Sigma-Aldrich	L-Citrulline-d7	C535703	Toronto Research Chemicals
L-Glutamine	49419	Sigma-Aldrich	L-Glutamine-d5	G597005	Toronto Research Chemicals
L-Glutamic acid	49449	Sigma-Aldrich	L-Glutamic acid-d5	G596963	Toronto Research Chemicals
L-Phenylalanine	78019	Sigma-Aldrich	L-Phenylalanine-d7	P319416	Toronto Research Chemicals
L-Tryptophan	T0254	Sigma-Aldrich	L-Tryptophan-d5	T947212	Toronto Research Chemicals
L-Tyrosine	T899975	Toronto Research Chemicals	L-Tyrosine-d2	H899977	Toronto Research Chemicals
L-Kynurenine	BIB6088	Apollo Scientific	L-Kynurenine-d4	K-008005	TLC Pharmaceutical Standards
Kynurenic acid	K660500	Toronto Research Chemicals	Kynurenic acid-d5	K660502	Toronto Research Chemicals
DL-Indole-3-lactic acid	I627100	Toronto Research Chemicals	DL-Indole-3-lactic acid-d5	I627102	Toronto Research Chemicals
Indole-3-acetic acid	I577344	Toronto Research Chemicals	Indole-3-acetic acid-d5	I577344	Toronto Research Chemicals
Indole-3-aldehyde	129445	Sigma-Aldrich	Indole-3-aldehyde-$^{13}C_8$	I614993	Toronto Research Chemicals
4-Hydroxyphenylpyruvic acid	114286	Sigma-Aldrich	4-Hydroxyphenylpyruvic acid-$^{13}C_9$	07491	Sigma-Aldrich
Phenylpyruvic acid	286958	Sigma-Aldrich	Phenylpyruvic acid-$^{13}C_9$	CLM-10604	Cambridge Isotope Laboratories

Table S2. Retention times and MRM transitions used for analyte and internal standard detection by LC-MS/MS.

Analyte	Separation Method	Ionization Mode	RT (min)	MRM-Transition (m/z)	Internal Standard	MRM-Transition (m/z)
L-Arginine	HILIC	Positive	7.2	175.2 → 70.1	L-Arginine-d7	182.2 → 77.1
L-Ornithine	HILIC	Positive	7.4	133.1 → 70.2	L-Ornithine-d6	139.0 → 76.2
L-Citrulline	HILIC	Positive	4.9	176.1 → 70.0	L-Citrulline-d7	183.2 → 77.2
L-Glutamine	HILIC	Positive	4.2	147.2 → 83.9	L-Glutamine-d5	152.1 → 89.1
L-Glutamic acid	HILIC	Positive	3.6	148.1 → 84.1	L-Glutamic acid-d5	153.1 → 87.9
L-Phenylalanine	HILIC	Positive	1.6	166.2 → 102.9	L-Phenylalanine-d7	172.9 → 108.1
L-Tryptophan	HILIC	Positive	1.6	205.0 → 118.2	L-Tryptophan-d5	209.9 → 122.1
L-Tyrosine	HILIC	Positive	2.2	182.1 → 91.0	L-Tyrosine-d2	184.0 → 92.0
L-Kynurenine	RPLC-1	Positive	0.8	208.8 → 94.1	L-Kynurenine-d4	213.1 → 97.9
Kynurenic acid	RPLC-1	Positive	1.0	190.0 → 162.1	Kynurenic acid-d5	194.9 → 167.1
DL-Indole-3-lactic acid	RPLC-1	Positive	1.7	206.0 → 118.2	DL-Indole-3-lactic acid-d5	211.1 → 122.0
Indole-3-acetic acid	RPLC-1	Positive	2.2	176.1 → 103.1	Indole-3-acetic acid-d5	181.2 → 107.0
Indole-3-aldehyde	RPLC-2	Negative	1.6	144.0 → 114.9	Indole-3-aldehyde-$^{13}C_8$	152.0 → 123.1
4-Hydroxyphenylpyruvic acid	RPLC-3	Negative	1.3	178.9 → 107.0	4-Hydroxyphenylpyruvic acid-$^{13}C_9$	188.0 → 114.1
Phenylpyruvic acid	RPLC-3	Negative	2.5	162.9 → 91.0	Phenylpyruvic acid-$^{13}C_9$	172.0 → 98.0

HILIC: hydrophilic interaction liquid chromatography; MRM: multiple reaction monitoring; m/z: mass-to-charge ratio; RPLC: reversed-phase liquid chromatography; RT: retention time.

Table S3. Amino acid, metabolite and IL4I1 concentrations in healthy donor and high-grade serous ovarian cancer patient samples. Amino acid and metabolite concentrations were determined by LC-MS/MS analysis. IL4I1 concentrations were determined by ELISA. Concentrations indicated in grey *italics* are deleteriously influenced by sample processing conditions.

	Arg (µM)			Orn (µM)			Cit (µM)			Gln (µM)			Glu (µM)		
	Plasma HD	Plasma OC	Ascites OC	Plasma HD	Plasma OC	Ascites OC	Plasma HD	Plasma OC	Ascites OC	Plasma HD	Plasma OC	Ascites OC	Plasma HD	Plasma OC	Ascites OC
Pt. 1		*66.8*			*189*			29.7			578			*207*	
Pt. 2		*29.2*			*106*			25.8			469			*132*	
Pt. 3		*32.0*	81.0		*67.9*	52.5		26.1	25.9		535	693		*168*	38.7
Pt. 4		*22.3*	79.8		*207*	43.3		46.5	22.5		576	640		*208*	83.4
Pt. 5		*35.9*	138		*120*	129		33.0	44.9		463	786		*85.8*	60.6
Pt. 6		*28.2*	141		*135*	104		22.5	28.0		406	613		*200*	91.3
Pt. 7		*14.2*	148		*147*	51.8		29.7	25.7		478	928		*107*	67.9
Pt. 8		*19.7*	63.2		*166*	51.5		23.6	18.4		407	507		*232*	33.1
Pt. 9		*65.2*	82.2		*103*	38.0		30.6	20.3		708	497		*103*	55.9
Pt. 10		*21.9*	49.1		*127*	98.5		20.3	24.2		425	466		*195*	224
Pt. 11		*9.70*	44.1		*120*	65.1		22.0	14.6		545	177		*187*	353
Pt. 12		*26.9*	32.3		*152*	112		24.5	21.0		473	401		*305*	545
Pt. 13		*17.9*	69.0		*141*	52.9		17.7	18.5		342	434		*197*	71.6
Pt. 14		*19.4*	96.3		*160*	54.2		26.3	13.1		524	569		*199*	68.2
Pt. 15		*39.6*	69.3		*129*	83.7		24.9	25.2		405	504		*301*	192
Pt. 16		*9.02*	70.1		*109*	48.8		16.7	16.9		397	479		*202*	282
Pt. 17		*35.5*	88.1		*151*	42.5		22.8	18.2		596	589		*290*	67.1
Pt. 18		*26.8*	91.9		*197*	59.0		32.2	18.2		467	537		*213*	46.1
Pt. 19		*47.4*	163		*106*	57.9		16.0	24.5		458	778		*127*	115
Pt. 20		*22.9*	122		*175*	54.9		31.1	28.5		399	519		*155*	83.2
Pt. 21		*22.4*	61.5		*133*	52.2		15.0	12.4		626	631		*186*	60.4
Pt. 22		*39.2*	105		*162*	69.0		19.5	17.9		415	528		*208*	93.4
Pt. 23		*16.4*	98.4		*129*	93.2		12.3	22.1		496	655		*140*	314
Pt. 24		*27.9*	101		*136*	59.9		21.1	21.8		418	540		*208*	160
Pt. 25			107			83.5			22.1			801			144
Pt. 26			108			49.6			23.6			671			95.6
Pt. 27			129			65.3			24.7			721			133
Pt. 28			144			40.6			22.0			762			119
Pt. 29			127			64.3			25.0			652			87.1
Pt. 30			166			81.1			30.0			691			289
Pt. 31			99.3			57.8			22.8			612			99.0
Pt. 32			118			70.4			32.2			658			105
Pt. 33			132			80.9			14.9			761			67.2
Pt. 34			139			63.3			29.4			570			150
HD 1	69.6			93.1			20.8			487			47.8		
HD 2	85.5			130			49.8			513			103		
HD 3	76.5			114			30.6			543			85.4		
HD 4	40.7			139			27.4			443			64.3		
HD 5	113			85.3			24.1			613			87.8		
HD 6	80.7			127			43.5			596			85.8		
HD 7	101			155			20.9			411			268		
HD 8	65.2			177			37.1			640			83.0		
HD 9	79.0			109			29.6			455			121		
HD 10	64.9			84.4			41.1			562			49.6		
HD 11	67.7			94.3			24.3			687			29.2		
HD 12	36.0			135			38.7			526			66.5		
HD 13	37.3			118			23.9			495			81.0		
HD 14	54.8			144			30.8			541			77.9		
HD 15	58.2			179			41.1			651			96.9		
HD 16	112			140			31.8			573			48.4		
HD 17	49.6			99.5			35.2			581			110		
HD 18	49.5			96.9			28.1			442			111		
HD 19	73.0			169			40.3			616			68.1		
HD 20	61.9			61.5			28.6			459			66.2		
HD 21	79.3			108			40.5			556			106		
HD 22	90.7			123			27.7			564			83.6		
HD 23	58.8			137			36.0			660			70.9		
HD 24	73.6			116			26.1			446			68.2		
HD 25	74.2			123			38.4			629			116		
HD 26	53.5			149			41.6			653			82.3		
HD 27	87.7			334			52.0			558			68.2		
HD 28	70.2			116			32.5			588			46.9		
HD 29	76.3			140			45.0			487			98.0		
HD 30	76.7			158			29.2			570			60.9		

Arg: L-arginine; Cit: L-citrulline; Gln: L-glutamine; Glu: L-glutamic acid; HD: healthy donor; OC: ovarian cancer patient; Orn: L-ornithine; Pt.: patient.

Table S3. *Cont.*

	Trp (μM)			Kyn (μM)			I3LA (nM)			I3AA (nM)			I3A (nM)			KynA (nM)		
	Plasma HD	Plasma OC	Ascites OC	Plasma HD	Plasma OC	Ascites OC	Plasma HD	Plasma OC	Ascites OC	Plasma HD	Plasma OC	Ascites OC	Plasma HD	Plasma OC	Ascites OC	Plasma HD	Plasma OC	Ascites OC
Pt. 1		41.1			2.32			503			1180			34.3			43.7	
Pt. 2		32.7			1.56			395			449			19.2			12.2	
Pt. 3		21.7	32.5		1.84	4.15		216	608		552	492		15.6	36.1		18.2	19.8
Pt. 4		37.3	32.3		2.22	4.01		738	552		1900	582		19.4	19.6		40.1	22.5
Pt. 5		29.6	58.8		1.32	4.21		267	1100		4130	3655		10.4	36.9		12.6	39.5
Pt. 6		26.0	54.7		1.80	3.10		928	490		1295	1415		31.7	45.0		45.1	29.0
Pt. 7		23.5	83.7		1.42	6.29		446	1445		1006	786		8.96	106		18.1	40.9
Pt. 8		27.4	31.9		1.44	5.27		397	546		278	482		22.0	27.8		23.0	31.8
Pt. 9		26.3	28.7		2.02	3.81		176	233		697	905		7.37	7.52		25.9	16.7
Pt. 10		21.4	24.6		4.21	6.18		436	463		475	592		12.7	27.1		58.4	43.4
Pt. 11		31.5	19.6		1.88	1.41		709	295		1815	1009		18.6	14.4		49.8	22.8
Pt. 12		30.7	27.0		2.02	3.33		765	518		863	721		20.9	15.7		33.4	29.2
Pt. 13		21.3	33.4		1.64	3.67		264	381		229	454		8.51	18.9		16.5	20.6
Pt. 14		32.3	29.0		2.12	3.08		417	217		1072	687		20.7	16.5		23.5	25.0
Pt. 15		49.9	35.6		2.46	5.15		950	1555		1410	899		32.7	52.8		14.3	26.8
Pt. 16		24.1	37.9		1.39	3.69		461	705		559	781		10.9	36.7		39.4	35.5
Pt. 17		22.1	31.0		2.20	2.98		250	223		978	705		9.01	9.41		16.1	12.5
Pt. 18		34.6	35.7		2.64	4.58		1725	1220		3445	1590		46.2	114		52.0	45.5
Pt. 19		52.6	73.7		3.72	8.27		639	941		1245	1450		32.2	43.5		27.5	52.8
Pt. 20		23.9	41.4		2.35	6.48		729	1020		382	781		15.2	67.6		45.4	36.1
Pt. 21		21.3	26.0		2.83	3.95		238	278		410	688		7.13	13.7		19.3	19.5
Pt. 22		21.7	38.5		1.51	4.36		182	365		532	1410		8.77	29.0		32.4	27.5
Pt. 23		26.6	31.8		1.13	2.67		318	423		772	698		16.3	21.5		20.0	37.7
Pt. 24		21.0	35.1		2.39	12.5		564	1030		199	452		18.9	57.2		51.0	85.7
Pt. 25			92.9			7.96			1355			855			46.7			41.0
Pt. 26			34.7			3.96			686			1240			31.0			37.1
Pt. 27			50.1			6.78			772			1290			37.5			37.0
Pt. 28			55.4			3.30			700			690			42.3			ND
Pt. 29			46.0			5.04			363			1275			33.3			31.5
Pt. 30			40.5			14.6			993			1023			66.7			128
Pt. 31			55.7			3.79			825			1035			48.6			32.4
Pt. 32			42.4			5.77			615			550			32.9			28.7
Pt. 33			56.0			3.67			492			1525			53.9			22.6
Pt. 34			48.8			6.19			348			1026			20.0			51.1
HD 1	60.1			1.62			887			1625			50.6			51.6		
HD 2	50.9			1.58			703			2235			36.0			38.4		
HD 3	45.7			1.56			762			1090			28.4			53.4		
HD 4	61.8			1.21			614			1040			45.4			23.7		
HD 5	76.4			2.14			862			1615			95.3			52.0		
HD 6	61.7			1.42			644			1005			37.7			24.3		
HD 7	64.4			1.83			545			949			36.7			33.5		
HD 8	68.0			2.40			665			1995			44.0			50.0		
HD 9	67.9			1.82			755			909			47.6			32.3		
HD 10	56.0			1.36			764			1055			42.1			20.8		
HD 11	33.9			1.45			286			2585			20.6			20.5		
HD 12	61.0			1.70			691			1465			35.9			29.5		
HD 13	57.8			1.44			669			1790			48.3			22.4		
HD 14	45.0			1.88			581			2985			30.9			21.0		
HD 15	60.6			1.90			964			1210			43.4			111		
HD 16	71.0			2.14			639			2030			49.0			26.6		
HD 17	57.0			1.87			810			5045			53.2			27.3		
HD 18	73.1			2.61			718			1735			56.0			21.8		
HD 19	62.4			1.95			809			1225			48.6			63.2		
HD 20	58.2			1.59			740			896			44.0			33.2		
HD 21	71.3			1.87			908			2015			86.0			32.6		
HD 22	58.3			2.57			1100			644			33.1			49.6		
HD 23	59.9			2.08			697			1895			55.5			27.3		
HD 24	65.7			2.42			740			1280			49.0			26.5		
HD 25	64.0			1.51			514			993			42.9			34.3		
HD 26	65.9			1.98			1075			2140			33.3			55.5		
HD 27	73.9			3.21			577			1290			36.9			49.2		
HD 28	67.7			2.04			1100			1520			57.2			66.2		
HD 29	70.9			3.05			766			1160			54.1			51.1		
HD 30	79.7			2.24			610			2350			48.1			37.0		

HD: healthy donor; I3A: indole-3-aldehyde; I3AA: indole-3-acetic acid; I3LA: indole-3-lactic acid; Kyn: L-kynurenine; KynA: kynurenic acid; OC: ovarian cancer patient; Pt.: patient; Trp: L-tryptophan.

Table S3. *Cont.*

	Phe (µM)			PP (nM)			Tyr (µM)			4HPP (nM)			IL4I1 (ng/mL)		
	Plasma HD	Plasma OC	Ascites OC	Plasma HD	Plasma OC	Ascites OC	Plasma HD	Plasma OC	Ascites OC	Plasma HD	Plasma OC	Ascites OC	Plasma HD	Plasma OC	Ascites OC
Pt. 1		77.0			*133*			78.1			*445*			< 62.5	
Pt. 2		55.8			*25.0*			52.3			*81.6*			150	
Pt. 3		46.4	74.6		*50.6*	350		34.3	67.1		*303*	1165		< 62.5	792
Pt. 4		76.0	68.4		*26.9*	202		89.3	71.5		*168*	738		498	1745
Pt. 5		83.7	147		*92.8*	360		64.6	127		*298*	1055		< 62.5	579
Pt. 6		72.8	114		*78.8*	776		65.9	131		*240*	1815		< 62.5	2896
Pt. 7		53.3	172		*26.2*	2435		54.8	158		*110*	3695		1812	7487
Pt. 8		80.5	77.6		*88.0*	446		60.5	59.1		*212*	873		< 62.5	1652
Pt. 9		74.7	69.4		*35.3*	180		67.6	72.2		*212*	488		< 62.5	112
Pt. 10		57.0	66.2		*30.0*	249		40.4	46.7		*90.6*	686		< 62.5	759
Pt. 11		65.5	46.2		*26.0*	29.5		58.0	41.4		*90.4*	146		< 62.5	156
Pt. 12		76.2	82.6		*56.8*	135		61.4	63.9		*128*	529		< 62.5	1560
Pt. 13		69.8	63.4		*26.8*	349		57.2	56.9		*85.9*	573		< 62.5	678
Pt. 14		64.7	84.4		*47.3*	185		59.4	79.9		*110*	692		< 62.5	730
Pt. 15		93.5	72.8		*222*	1035		77.7	58.5		*355*	1255		< 62.5	1189
Pt. 16		50.1	70.2		*47.6*	393		39.0	52.9		*114*	794		< 62.5	1489
Pt. 17		47.4	55.3		*19.1*	126		48.8	55.9		*84.4*	345		< 62.5	251
Pt. 18		75.4	74.8		*171*	6255		88.7	76.5		*424*	13100		1364	4995
Pt. 19		69.4	119		*120*	987		65.1	110		*313*	1635		< 62.5	2836
Pt. 20		62.9	84.0		*38.3*	821		46.9	69.5		*89.0*	784		< 62.5	2578
Pt. 21		73.3	70.9		*27.3*	163		55.1	59.4		*94.9*	631		< 62.5	499
Pt. 22		92.5	105		*33.6*	782		72.6	83.3		*127*	905		< 62.5	647
Pt. 23		75.5	64.2		*41.5*	50.9		55.2	66.0		*92.8*	413		< 62.5	2100
Pt. 24		51.7	58.2		*20.8*	843		54.8	64.6		*107*	1355		< 62.5	3172
Pt. 25			196			944			159			978			848
Pt. 26			87.1			325			82.3			1095			919
Pt. 27			98.6			595			105			1065			1601
Pt. 28			126			900			112			1115			148
Pt. 29			93.9			311			82.6			528			4103
Pt. 30			129			4560			104			3945			1315
Pt. 31			81.9			327			78.8			771			540
Pt. 32			81.5			340			75.8			871			855
Pt. 33			111			783			123			2285			1378
Pt. 34			92.7			64.8			95.2			509			892
HD 1	76.6			249			60.4			349			< 62.5		
HD 2	66.3			101			58.7			274			< 62.5		
HD 3	76.3			111			57.3			250			< 62.5		
HD 4	68.0			121			67.4			262			1514		
HD 5	81.8			192			120			953			< 62.5		
HD 6	68.3			103			63.5			310			697		
HD 7	87.9			128			91.4			152			< 62.5		
HD 8	64.6			104			91.6			360			< 62.5		
HD 9	79.9			154			81.2			361			< 62.5		
HD 10	46.5			116			51.4			358			< 62.5		
HD 11	42.2			101			35.3			422			< 62.5		
HD 12	66.8			134			83.6			491			< 62.5		
HD 13	67.0			125			55.6			269			< 62.5		
HD 14	47.6			82.6			51.3			258			< 62.5		
HD 15	69.7			82.5			93.9			395			126		
HD 16	76.4			150			93.4			645			< 62.5		
HD 17	72.1			167			69.8			423			< 62.5		
HD 18	90.2			251			109			654			< 62.5		
HD 19	88.7			146			82.1			443			< 62.5		
HD 20	69.0			102			77.5			465			497		
HD 21	72.1			144			92.1			787			< 62.5		
HD 22	68.8			131			87.4			454			< 62.5		
HD 23	81.8			147			86.8			446			< 62.5		
HD 24	78.8			177			86.6			589			< 62.5		
HD 25	69.6			104			62.4			314			< 62.5		
HD 26	68.0			106			82.2			413			< 62.5		
HD 27	115			147			142			564			< 62.5		
HD 28	62.0			144			71.0			470			< 62.5		
HD 29	79.1			174			111			899			< 62.5		
HD 30	97.3			154			142			617			< 62.5		

4HPP: 4-hydroxyphenylpyruvic acid; HD: healthy donor; IL4I1: interleukin 4 induced 1; OC: ovarian cancer patient; Phe: L-phenylalanine; PP: phenylpyruvic acid; Pt.: patient; Tyr: L-tyrosine.

Table S4. IL4I1 metabolite and enzyme concentrations in pleural effusion samples from non-small cell lung cancer patients. PP and 4HPP concentrations were determined by LC-MS/MS analysis. IL4I1 concentrations were determined by ELISA.

	**PP (nM)**	**4HPP (nM)**	**IL4I1 (ng/mL)**
Pt. 1	1200	3005	4070
Pt. 2	367	174	2539
Pt. 3	368	1490	4035
Pt. 4	12.1	87.5	234
Pt. 5	279	228	1018
Pt. 6	33.5	74.7	62.0
Pt. 7	1030	428	868
Pt. 8	91.9	118	62.0
Pt. 9	354	508	1307
Pt. 10	80.0	208	3254
Pt. 11	391	223	341
Pt. 12	273	125	691
Pt. 13	25.2	108	525
Pt. 14	460	368	672
Pt. 15	251	329	589
Pt. 16	1480	482	643
Pt. 17	223	130	62.0
Pt. 18	531	100	115
Pt. 19	202	62.5	489
Pt. 20	509	290	62.0
Pt. 21	413	153	1968
Pt. 22	2990	1850	2459
Pt. 23	595	231	630
Pt. 24	1885	840	883

4HPP: 4-hydroxyphenylpyruvic acid; IL4I1: interleukin 4 induced 1; PP: phenylpyruvic acid.

Table S5. Clinicopathological characteristics and details on the treatment of included high-grade serous ovarian cancer patients who completed primary treatment and subgroups thereof.

		**Subgroups Based on Collected Samples ***		
	**All Patients with Completed Treatment (*n* = 26)**	**Patients with Plasma Collected (*n* = 20)**	**Patients with Ascites Collected (*n* = 24)**	**Patients with Both Plasma and Ascites Collected (*n* = 18) †**
Age (years)				
Median (IQR)	60.5 (57–67)	60 (54–64)	61.5 (58–67)	61 (57–65)
BMI (kg/m²)				
Median (IQR)	26 (22–28)	24 (22–27)	26 (22–29)	24 (22–27)
FIGO stage				
II	2 (7.7%)	1 (5.0%)	2 (8.3%)	1 (5.6%)
III	17 (65.4%)	13 (65.0%)	15 (62.5%)	11 (61.1%)
IV	7 (26.9%)	6 (30.0%)	7 (29.2%)	6 (33.3%)
Surgery				
PDS	11 (42.3%)	10 (50.0%)	10 (41.7%)	9 (50.0%)
IDS	11 (42.3%)	7 (35.0%)	10 (41.7%)	6 (33.3%)
IDS/HIPEC	4 (15.4%)	3 (15.0%)	4 (16.7%)	3 (16.7%)
Outcome surgery				
Optimal	8 (30.8%)	7 (35.0%)	8 (33.3%)	7 (38.9%)
Complete	18 (69.2%)	13 (65.0%)	16 (66.7%)	11 (61.1%)
Chemotherapy				
Carboplatin + paclitaxel	24 (92.3%)	19 (95.0%)	22 (91.7%)	17 (94.4%)
Other regimen	2 (7.7%)	1 (5.0%)	2 (8.3%)	1 (5.6%)
Platinum sensitive ‡				
Yes	22 (84.6%)	16 (80.0%)	20 (83.3%)	14 (77.8%)
No	4 (15.4%)	4 (20.0%)	4 (16.7%)	4 (22.2%)

BMI: body mass index; FIGO: International Federation of Gynecology and Obstetrics; IDS: interval debulking surgery; IDS/HIPEC: interval debulking surgery in combination with hyperthermic intraperitoneal chemotherapy; IQR: interquartile range; PDS: primary debulking surgery. Percentages may not total to 100% due to rounding. * Samples from remaining patients were missing for logistic reasons. † Also referred to as "overlapping patients". ‡ Platinum sensitivity is defined as a progression-free survival greater than six months.

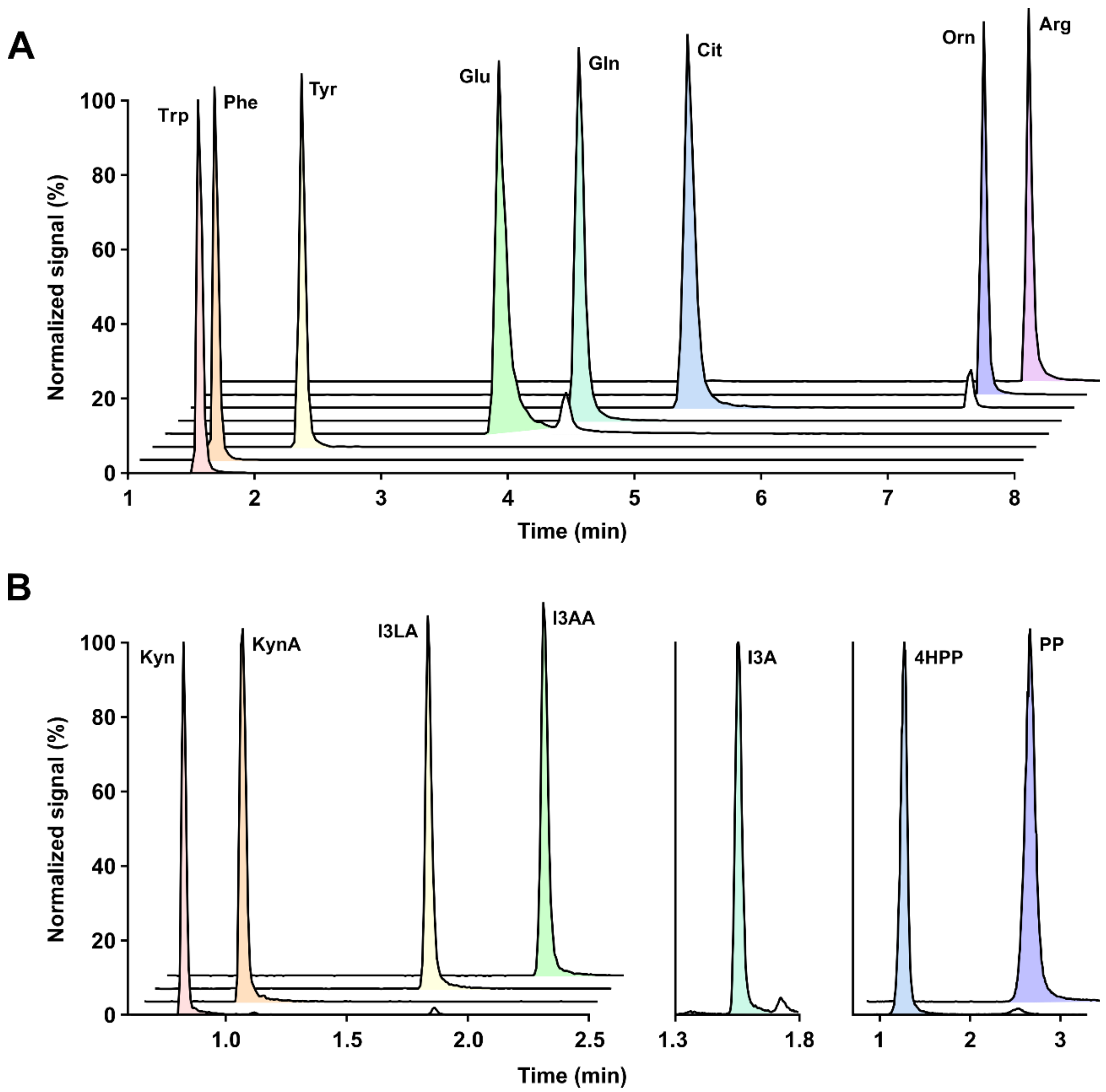

Figure S1. Representative chromatograms for analyte separation in surrogate matrices by (**A**) hydrophilic interaction liquid chromatography (HILIC)- and (**B**) reversed-phase liquid chromatography (RPLC)-tandem mass spectrometry (MS/MS). 4HPP: 4-hydroxyphenylpyruvic acid; Arg: L-arginine; Cit: L-citrulline; Gln: L-glutamine; Glu: L-glutamic acid; I3A: indole-3-aldehyde; I3AA: indole-3-acetic acid; I3LA: indole-3-lactic acid; Kyn: L-kynurenine; KynA: kynurenic acid; Orn: L-ornithine; Phe: phenylalanine; PP: phenylpyruvic acid; Trp: L-tryptophan; Tyr: L-tyrosine.

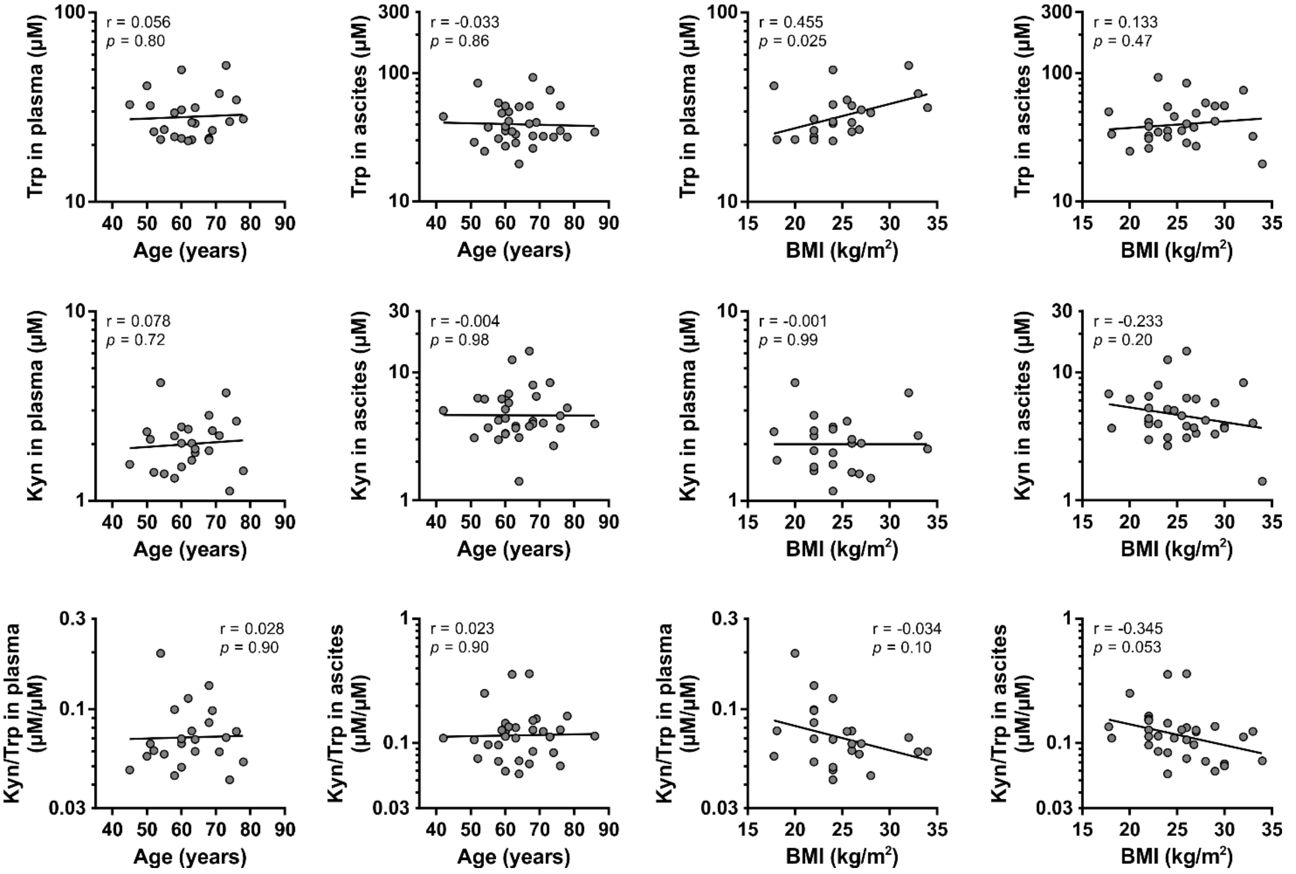

Figure S2. Correlation between Trp levels, Kyn levels or Kyn/Trp ratios in plasma (n = 24) or ascites (n = 32) and age or BMI of high-grade serous ovarian cancer patients. Results of Pearson's correlation analyses are indicated in the graphs. BMI: body mass index; Kyn: kynurenine; r: Pearson's correlation coefficient; Trp: tryptophan.

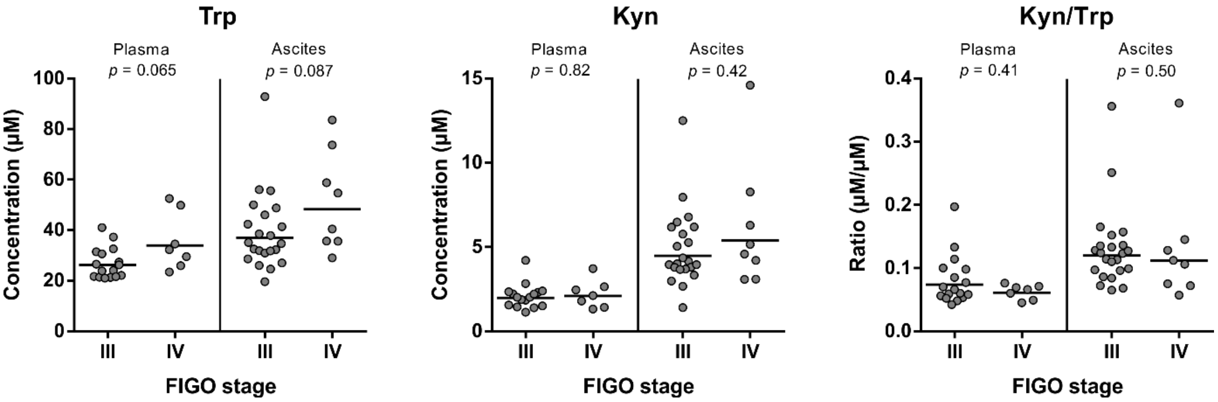

Figure S3. Comparison of Trp levels, Kyn levels and Kyn/Trp ratios in high-grade serous ovarian cancer patients with stage III (n = 16 for plasma; n = 22 for ascites) and stage IV disease (n = 7 for plasma; n = 8 for ascites). Horizontal lines indicate geometric means. Results of unpaired, two-tailed Mann–Whitney U tests are indicated in the graphs. Since only two patients with stage II disease were included in the study, these patients were excluded from this analysis. FIGO: International Federation of Gynecology and Obstetrics; Kyn: L-kynurenine; Trp: L-tryptophan.

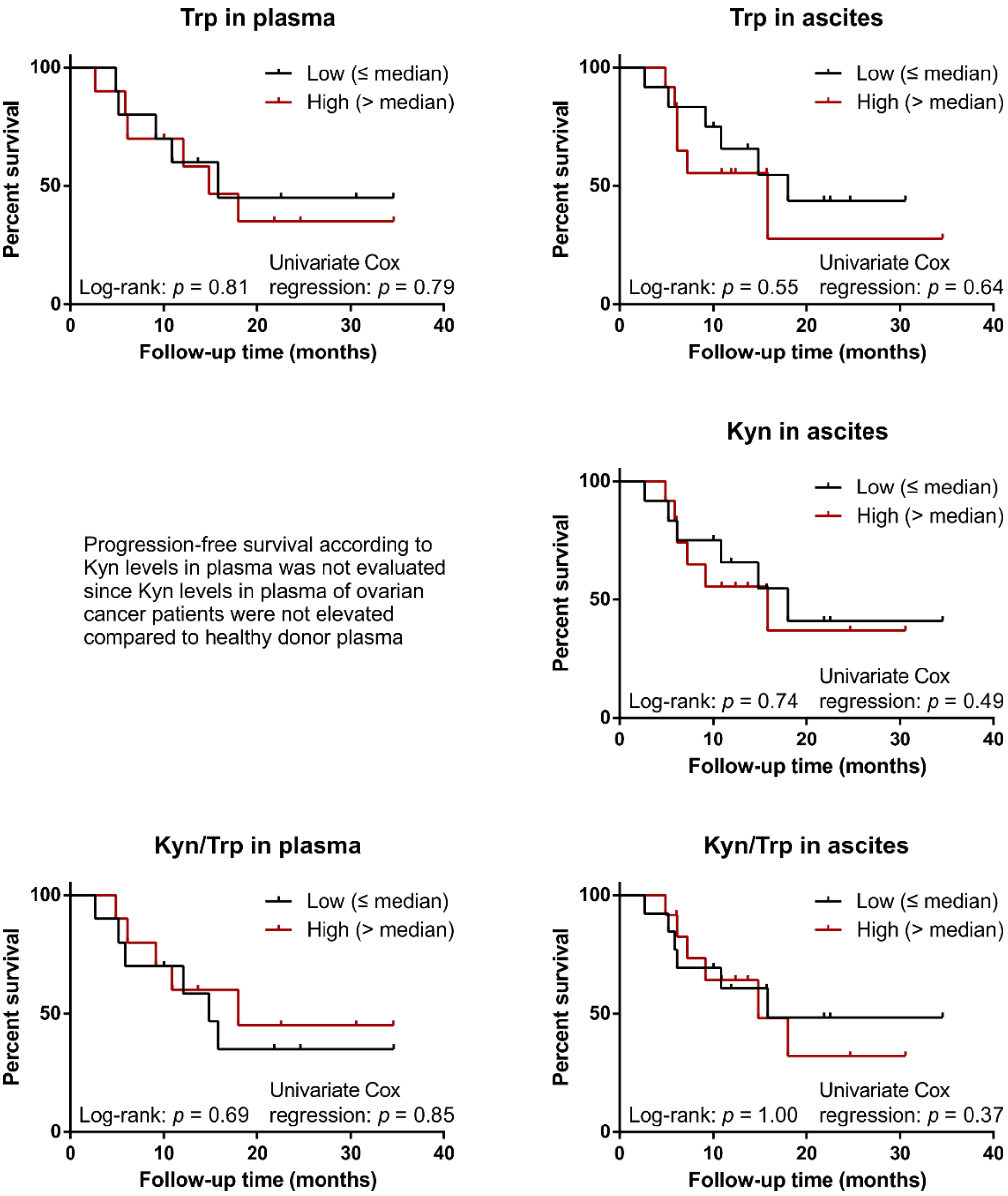

Figure S4. Kaplan–Meier progression-free survival curves according to Trp levels, Kyn levels and Kyn/Trp ratios in plasma or ascites of high-grade serous ovarian cancer patients who completed treatment (n = 20 for plasma; n = 24 for ascites). Results of log-rank and univariate Cox regression analyses are indicated in the graphs. Multivariate regression analysis was not performed due to small sample sizes. Kyn: L-kynurenine; Trp: L-tryptophan.

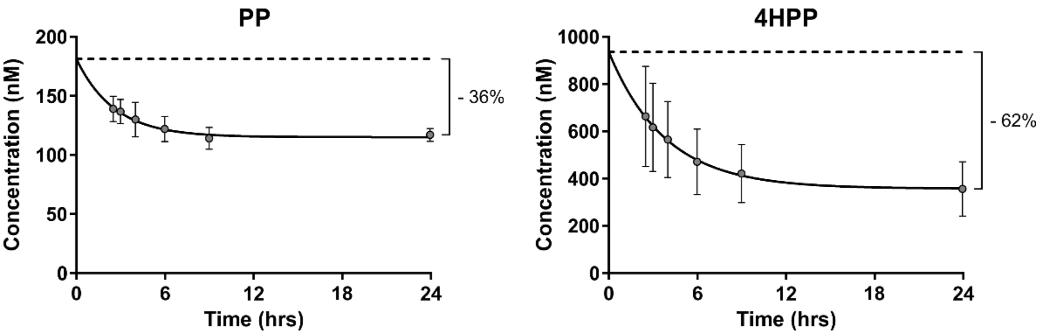

Figure S5. Estimation of the reduction in PP and 4HPP levels in healthy donor blood stored at room temperature prior to plasma separation. Data have been fitted with an exponential one-phase decay curve to estimate the reduction in PP and 4HPP levels between the time of blood collection (t = 0 hours) and the approximated time at which the ovarian cancer patient plasma samples were processed (t = 24 hours). 4HPP: 4-hydroxyphenylpyruvic acid; PP: phenylpyruvic acid.

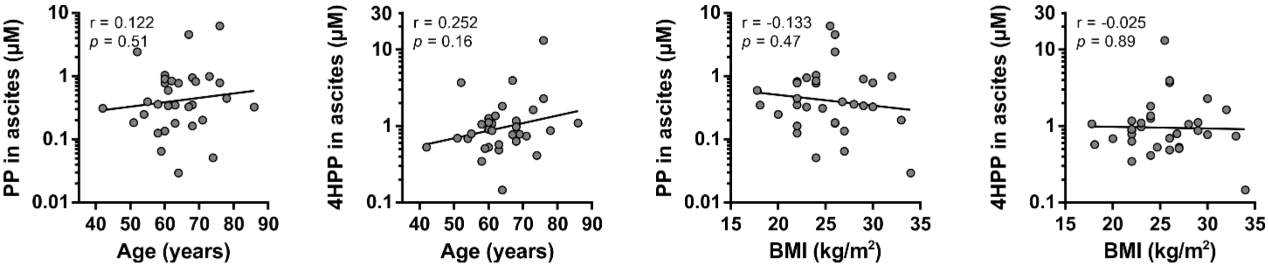

Figure S6. Correlation between PP or 4HPP levels in ascites and age or BMI of high-grade serous ovarian cancer patients (*n* = 32). Results of Pearson's correlation analyses are indicated in the graphs. BMI: body mass index; 4HPP: 4-hydroxyphenylpyruvic acid; PP: phenylpyruvic acid; r: Pearson's correlation coefficient.

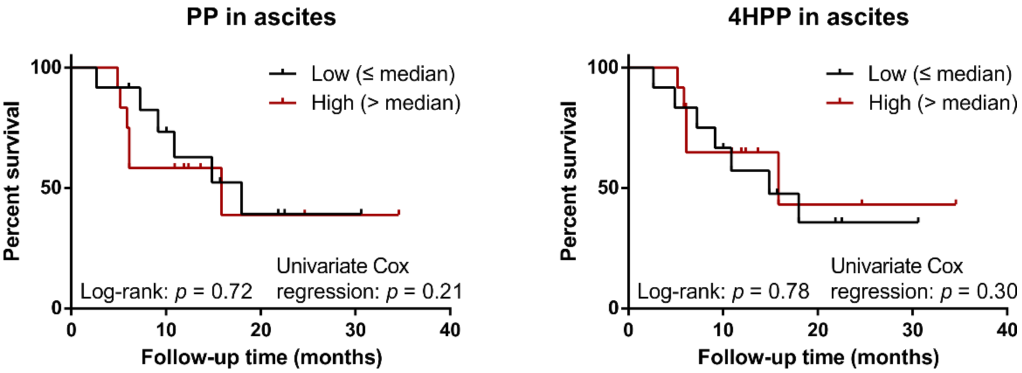

Figure S7. Kaplan–Meier progression-free survival curves according to PP and 4HPP levels in ascites of high-grade serous ovarian cancer patients who completed treatment (*n* = 24). Results of log-rank and univariate Cox regression analyses are indicated in the graphs. Multivariate regression analysis was not performed due to small sample sizes. 4HPP: 4-hydroxyphenylpyruvic acid; PP: phenylpyruvic acid.
